# Forearm bone mineral density as a predictor of reduction loss in distal radius fractures treated with cast immobilization

**DOI:** 10.3389/fsurg.2022.1043002

**Published:** 2022-11-28

**Authors:** Sung Tan Cho, Jin Hwan Kim, Sung San Lee, Yong Jae Lee, Hyun Il Lee

**Affiliations:** Department of Orthopaedic Surgery, Inje University Ilsan Paik Hospital, Goyang-si, Gyeonggi-do, South Korea

**Keywords:** forearm, osteoporosis, distal radius fracture, reduction loss, bone mineral density

## Abstract

**Objective:**

Many potential predictors have been identified and proposed for predicting late reduction loss in distal radius fractures. However, no report exists on whether the bone mineral density (BMD) of the forearm correlates with the loss of reduction in distal radius fractures. This study aimed to investigate whether forearm BMD can be used as a predictor of reduction loss in distal radius fractures treated with cast immobilization.

**Methods:**

Ninety patients with distal radius fractures were divided into two groups according to the maintenance or loss of reduction evaluated from radiographs taken at least 6 weeks after their injury. Lumbar and forearm BMD (total and metaphysis) T-scores were measured and compared between the maintenance of reduction (MOR) group and the loss of reduction (LOR) group. Additionally, serologic markers (C-terminal telopeptide, osteocalcin, vitamin D) and radiologic risk factors (intra-articular fracture, ulnar fracture, dorsal comminuted fracture, volar hook) were evaluated and a logistic multiple regression analysis was performed to know the main risk factors of reduction loss.

**Results:**

Reduction loss was observed in 38 patients (42.2%). The total and metaphyseal BMD of the forearm was less in the LOR group than in the MOR group. However, the difference was not statistically significant [−2.9 vs. −2.5 for total (*p* = 0.18), −2.3 vs. −2.0 for metaphysis (*p* = 0.17)]. Multiple logistic regression analysis showed initial dorsal comminution (*p* = 0.008) and ulnar variance (*p* = 0.01) were the main risk factors for reduction loss.

**Conclusions:**

Forearm BMD was not a valuable prognostic factor for reduction loss in distal radius fractures. Initial dorsal comminution and ulnar variance rather than forearm BMD should be considered preferentially when predicting which patients are at high risk of reduction loss in distal radius fractures.

## Introduction

Distal radius is the most common fracture site of the forearm (1). Patients with distal radius fractures initially undergo closed reduction and splinting with close radiographic follow-up in the first 1–2 weeks post-treatment to verify the maintenance of reduction. However, if the likelihood of collapse is high, it may be advisable to recommend early surgery for such patients. Unfortunately, there are still controversies regarding which factors are significant risk factors of failure on a conservative treatment for distal radius fractures (2–5). Many potential predictors have been identified and proposed for predicting late reduction loss. For example, demographic factors such as age, sex, and radiographic parameters such as initial displacement, degree of dorsal comminution, presence of an intra-articulate fracture or ulnar fracture, and loss of the volar hook have been suggested (3–5). However, a recent meta-analysis showed that only dorsal comminution and age were reliable predictors of reduction loss (6).

The bone density of the lumbar spine and hips is usually used as the standard measurement for osteoporosis, but these areas have shortcomings in that they do not reflect the local bone density relevant to fractures in other parts of the body. In fact, it has also been reported that the stability of distal radius fractures and the bone density of the vertebrae and hips in patients are not statistically related (7).

To the best of our knowledge, no report exists on whether the bone density of the local bone correlates with the loss of reduction performed in the distal radius. We estimate that osteoporosis can be used as a prognostic factor because older patients or patients with extensive dorsal comminutions are associated with a more severe form of osteoporosis. The purpose of this study was to determine whether the bone mineral density (BMD) of the forearm in patients with distal radius fractures is correlated with loss of reduction. We hypothesized that the BMD of the forearm was inversely related to the loss of reduction after closed manipulation.

## Materials and methods

This retrospective study was approved by our institutional review board and conducted in accordance with the Declaration of Helsinki. The study included patients who visited our hospital between March 2016 and June 2018 for distal radius fractures and received a short arm cast as a conservative treatment after acceptable reduction. Written informed consent was obtained from all the patients. A standard definition of acceptable reduction has not been established yet. Therefore, the authors attempted to define acceptable reduction by referring to some of the definitions used previously (8–11). As a result, acceptable reduction was defined as (a) radial inclination ≥10°, (b) −10° ≤ volar tilt ≤20°, and (c) ulnar variance ≤3 mm. The inclusion criteria were as follows: (a) Acceptable reduction after initial manipulation, (b) age 50 years or older, (c) having undergone BMD testing of the forearm (total and metaphysis) and lumbar spine within 3 months from the date of injury, (d) having followed up for observation with radiographs for at least 6 weeks. The exclusion criteria were: (a) Non-displaced distal radius fracture, (b) patients who had fractures in both arms and whose forearm BMD could not be obtained due to volar plates on the wrist of the other side, and (c) unacceptable reduction regardless of whether the operation was performed or not. Finally, a total of 90 patients were enrolled in the study.

**Figure 1:**
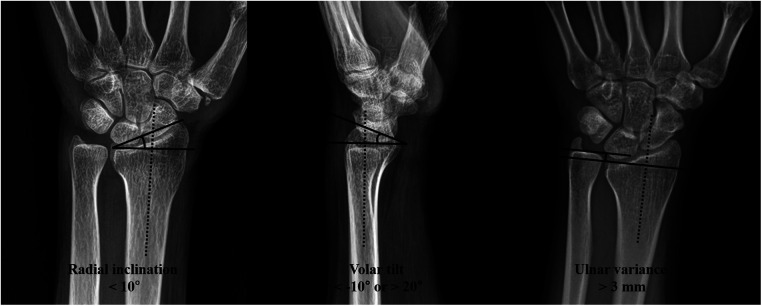
Radiological evaluation of the distal radius. If radial inclination, volar tilt, or ulnar variance of the patients fell within the range indicated below, they were included in the loss of reduction (LOR) group.

The patients were divided into two groups according to whether they experienced reduction loss or not, based on simple radiographs (anteroposterior and lateral views) taken at least six weeks after injury. All radiographs were taken by a radiologist and reduction loss was defined as the opposite of acceptable reduction. The definition was as follows: (a) Radial inclination <10°, (b) volar tilt <−10° or >20°, or (c) ulnar variance >3 mm (Figure 1). Patients with reduction loss were included in the loss of reduction (LOR) group, and those without reduction loss were classified as the maintenance of reduction (MOR) group.

**Figure 2:**
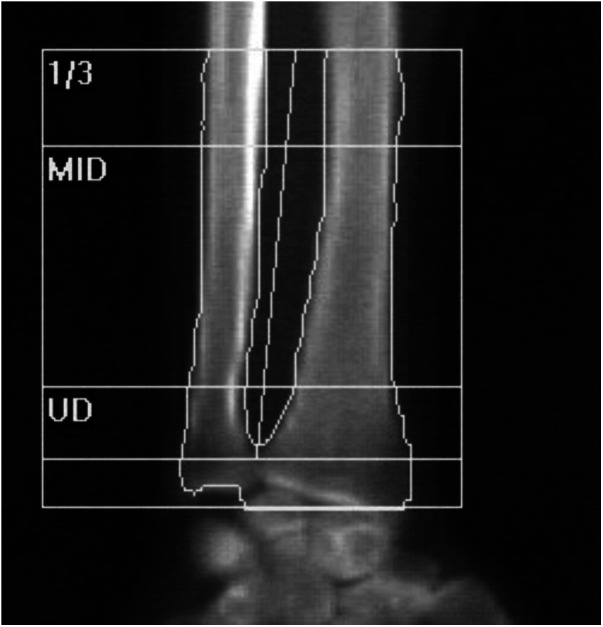
Regions of the ulna on forearm densitometry. UD, ultradistal (metaphysis); MID, middistal, 1/3; proximal.

The BMDs of the lumbar spine and forearm were measured using Horizon Wi (software version 13.6.0.2) from Hologic (Marlborough, Massachusetts, United States of America). The forearm BMD was measured as the total forearm and the metaphysis of forearm. These regions were identified using the analysis software supplied by the manufacturer (Figure 2). The T-score was calculated based on the data of Asian women provided by Hologic, according to the manufacturer's guidelines.

In this study, serologic markers representing bone turnover were also measured. The C-terminal telopeptide for bone resorption and osteocalcin for bone formation were measured together with BMD (12). In addition, the level of vitamin D, which is known to increase BMD and reduce the risk of fractures, was measured to determine whether there was a correlation with reduction loss (13).

The presence of intra-articular fractures, ulnar fractures, dorsal comminuted fractures, and volar hooks (14), which are radiologic risk factors for reduction loss, were evaluated altogether using radiographs taken pre- and post-reduction. When the fracture pattern was ambiguous, computed tomography was additionally taken to confirm the presence of these risk factors.

To determine the appropriate sample size, the author compared 20 patients in the LOR group with 20 patients in the MOR group in the pilot study. The results of the pilot study showed that the total forearm BMD T-score was −2.2 and −3.0 in the MOR and LOR groups, respectively. The standard deviation was 1.1. With these results, the appropriate number of samples was determined to be 30 per group when the power and significance levels were set at 80% and 0.05, respectively. Therefore, the inclusion of a total of 90 patients (38 patients for LOR and 52 patients for MOR) in the study was thought to be adequate.

Parametric statistics were used for the normally distributed variables of the two groups. Otherwise, non-parametric statistics were used. Comparisons between each group of continuous variables were made using the independent samples *t*-test or paired *t*-test. For nominal variables, Fisher's exact test or the *χ*^2^ test was used. Using the variables with a significance of <0.2, a multivariate analysis was performed using logistic multiple regression analysis. The best set of variables predicting outcomes was obtained using stepwise multiple logistic regression analysis. A *p*-value of <0.05 was considered statistically significant.

## Results

Reduction loss was observed in 38 patients (42.2%), while 52 patients (57.8%) showed adequate fracture union without reduction loss. After discussion with patients, four patients in the LOR group had undergone surgical treatment to prevent possible problems such as pain and functional disability during the course of conservative treatment. The LOR and MOR groups were compared according to age, sex, the BMD of the lumbar spine and forearm, serologic markers for osteoporosis, and radiologic risk factors. Age and sex did not show any statistically significant difference between the two groups (Table 1).

**Table 1:** Comparison between the maintenance of reduction (MOR) group and the loss of reduction (LOR) group.

	MOR	LOR	*p*-value
Age	65.3	67.7	0.22^a^
Sex (Female %)	92.3	97.4	0.30^b^
Initial RI (°)	20.9	18.01	0.07^a^
Initial VT (°)	−6.2	−3.0	0.44^a^
Initial UV (mm)	1.5	2.3	0.003^a^
Lumbar BMD (T-score)	−1.7	−1.9	0.41^a^
Forearm BMD, Total (T-score)	−2.5	−2.9	0.18^a^
Forearm BMD, metaphysis (T-score)	−2.0	−2.3	0.17^a^
Vitamin D level (ng/mL)	18.8	18.8	0.99
C-terminal telopeptide (ng/mL)	0.32	0.31	0.74
Osteocalcin (ng/mL)	6.25	8.57	0.25

MOR, maintenance of reduction group; LOR, loss of reduction group; RI, radial inclination; VT, volar tilt; UV, ulnar variance; BMD, bone mineral density.

^a^ T-test.

^b^ *χ*^2^.

In general, the forearm BMD was significantly lower than the lumbar spinal BMD (Table 2). The total forearm BMD of the LOR group was −2.9, which was lower than that of the MOR group (−2.5) (*p* = 0.18). In addition, the BMD of the forearm metaphysis was −2.3 in the LOR group and −2.0 in the MOR group (*p* = 0.17). However, both results showed no statistically significant differences between the two groups. In addition, there was no significant difference in the lumbar BMD between the MOR (−1.7) and LOR (−1.9) groups (*p* = 0.41) (Table 1).

**Table 2:** Comparison between forearm BMD and lumbar BMD.

	Lumbar	Forearm total	Forearm metaphysis
BMD (T-score)	−1.8	−2.7	−2.1
*p*-value^a^ (Lumbar)	–	<0.001	0.015
*p*-value^a^ (Forearm total)	<0.001	–	<0.001

BMD, bone mineral density.

^a^ Paired *t*-test.

The serologic markers (vitamin D, C-terminal telopeptide, and osteocalcin) that reflect bone metabolism showed no statistically significant differences between the two groups (Table 1).

When measuring the degree of initial displacement (radial inclination, volar tilt, and ulnar variance), the mean of the initial ulnar variance was 1.5 mm in the MOR group and 2.3 mm in the LOR group, showing a statistically significant difference (*p* = 0.003). The initial radial inclination and volar tilt were not significantly different between the two groups (Table 1).

**Figure 3:**
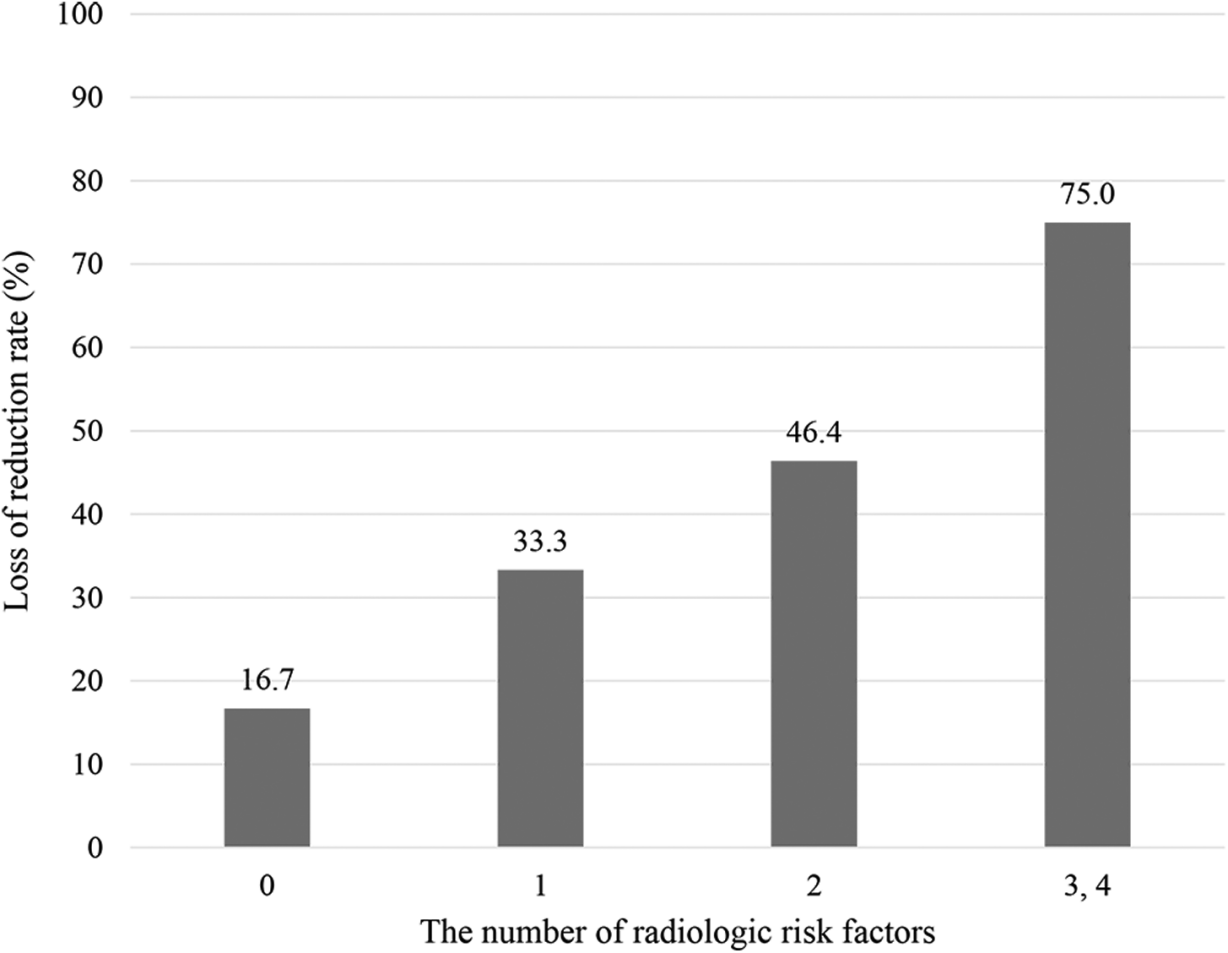
Loss of reduction rate according to radiologic risk factors. The horizontal axis indicates the number of radiologic risk factors.

The LOR group had a higher probability of intra-articular fractures, dorsal comminuted fractures, ulnar fractures, and volar hooks (Table 3). All variables, except for ulnar fractures, showed statistically significant differences between the MOR and LOR groups. Furthermore, the higher the number of radiologic risk factors, the higher the rate of reduction loss (Figure 3).

**Table 3:** Comparison of the presence of radiologic risk factors between the two groups.

	MOR	LOR	*p*-value^a^
Intra-articular fracture (%)	13.5	36.8	0.01
Ulnar fracture (%)	28.8	47.4	0.072
Dorsal comminution (%)	36.5	68.4	0.003
Volar hook (%)	32.7	55.3	0.032

MOR, maintenance of reduction group; LOR, loss of reduction group.

^a^ *χ*^2^.

We also investigated whether the local BMD of the forearm (metaphysis) is related to the radiologic risk factors for reduction loss (intra-articular fracture, ulnar fracture, dorsal comminution, and volar hook). However, it was found that osteoporosis of the local forearm was not statistically related to the presence of radiologic risk factors.

For multiple logistic regression analysis, we used variables with a significance of <0.2 (initial radial inclination, initial ulnar variance, forearm BMD, intra-articular fracture, ulnar fracture, dorsal comminution, and volar hook) in the multiple logistic regression analysis. The best set of variables predicting reduction loss was determined using stepwise multiple logistic regression analysis. In conclusion, among many factors, initial dorsal comminution (*p* = 0.008) and ulnar variance (*p* = 0.01) were the main risk factors for reduction loss (Table 4).

**Table 4:** Result of reduction loss-related factors from logistic regression analysis.

	Odds ratio	95% CI	*p*-value^a^
Initial ulnar variance	1.698	1.134–2.544	0.010
Dorsal comminution	3.529	1.398–8.912	0.008

^a^ Logistic regression analysis.

## Discussion

This study aimed to determine whether the local BMD of the forearm is a useful tool for predicting reduction loss in distal radius fractures. Based on the results, local forearm BMD was not related to reduction loss of distal radius fractures. Instead, it was found that initial dorsal comminution and ulnar variance contributed to the reduction loss. Considering that initial dorsal comminution or ulnar variance affects reduction loss more than serologic markers or BMD, it should be interpreted that the stability of the gross structure rather than the microstructure is more important for maintaining reduction.

Until recently, there have been several studies addressing the loss of reduction in distal radius fractures, but clear prognostic factors have yet to be determined (15–17). A study by Mackeneey et al. (18) estimated the possibility of future reduction loss using age, degree of comminution, and potential of dorsal deviation. However, further studies have shown that this may not be accurate. A systematic review conducted by Walenkamp et al. (6) documented that the presence of a dorsal comminuted fracture, age (60 years and older), and female sex were related to reduction loss in distal radius fractures. However, the use of dorsal comminution as a parameter for predicting reduction loss in the conservative treatment of distal radius fractures is limited in actual situations. This is because the inter-observer accuracy is low and there is no clear definition of dorsal comminution. In addition, there is a limitation in the clinical use of age and sex to predict the loss of reduction. Compared to young patients, elderly patients with fewer functional demands are more likely to tolerate loss of reduction. Therefore, surgery is more frequently performed in younger patients than in elderly patients, even if reduction loss occurs (19, 20). Therefore, selecting a patient for surgery based on age is not reasonable. Furthermore, since most osteoporotic fractures occur in women, sex is not useful in determining which patients require early surgical intervention. For these reasons, we tried to identify additional risk factors such as forearm BMD. However, our study showed that there was no significant difference of forearm BMD between LOR and MOR groups, which was thought to represent local bone condition of distal radius.

Instead of forearm BMD, there have been studies on the relationship between hip BMD and the prognosis of distal radius fractures. Clayton et al. (21) documented the relationship between osteoporosis and the prognosis of distal radius fractures and showed that a decrease in the BMD of the hip was correlated with more early instability, later carpal alignment, and malunion of the distal radius. It revealed that patients with osteoporosis had a 43% probability of early instability, a 39% probability of having late carpal alignment, and a 66% probability of having malunion after conservative treatment of distal radius fractures. In contrast, patients with normal BMD had a 28% probability of early instability, a 25% probability of late carpal malalignment, and a 48% probability of malunion. In addition, the study by Webber et al. (22) proved that the bicortical thickness of the distal radius was positively correlated with femoral BMD but not with lumbar spinal BMD. Compared with the axial spine, the femur may show more similarities in bone quality as appendicular bones. The importance of hip BMD in forearm fractures is further highlighted by Yoda et al. (23) They investigated the association of the ulnar fracture in distal radius fracture with the BMD of the hip, the lumbar spine, and the forearm (metaphysis). The study revealed that only the BMD of the hip was significantly different between the ulnar fracture group and the non-ulnar fracture group (23). Even though the hip BMD was not measured in our study, it seems to be more important than forearm BMD in predicting reduction loss in distal radius fractures. Therefore, a prospective study with more number of patients is needed in the future.

According to the osteoporosis fracture cascade, the forearm is the earliest site to respond to injury (24). The fact that forearm fractures usually occur earlier than vertebral or hip fractures in postmenopausal women may be related to lower forearm BMD levels (24–26). Previous studies revealed that osteoporosis is more obvious and, thus, more easily detected in the peripheral regions (forearm) than in the central regions (spine and hip) (26, 27). Miyamura et al. (27) analyzed the forearm BMD in postmenopausal women. In their study, the postmenopausal women with distal radius fractures had significantly lower forearm BMD than the postmenopausal women with no history of distal radius fractures (27). However, the spine and hip measurements did not differ significantly between the two groups. In another previous study comparing bone densitometry of the spine, hip, and forearm in postmenopausal women, the BMD values were −1.79, −1.69, and −2.58, respectively (26). The wrist BMD was significantly lower than that of the spine and hip regions. In our study, although hip joint BMD was not measured, forearm BMD was significantly lower than lumbar BMD (Table 2). However, considering that the forearm BMD was not significantly different between the MOR and LOR group in our study, it is not reasonable to use the forearm BMD as a predictor of reduction loss in distal radius fracture, even though it showed the decreased BMD more prominently than that of the spine.

The strong point of this study is that we analyzed the local BMD of the forearm for the first time as a factor of reduction loss in distal radius fracture. However, this study is not free from several limitations as follows: First, the T-score was based on the data on Asian women provided by the inspection equipment manufacturer for bone density of the forearm, not Korean women. Second, hip BMD was not measured in this study. Since hip BMD is a useful indicator of cortical bone quality, it could better reflect the bone density state of the distal radius than lumbar BMD. Last, even if the appropriate number of sample size was determined, future study with a greater number of patients is needed to improve the statistical outcomes.

In conclusion, forearm BMD was not related to reduction loss of distal radius fractures. For the prediction of reduction loss in distal radius fractures, initial dorsal comminution and ulnar variance should be considered more than forearm BMD. Furthermore, in case of more radiologic risk factors, caution is warranted not to miss the appropriate time for surgical intervention.

## Contribution to the field statement

Patients with distal radius fractures initially undergo closed reduction and splinting with close radiographic follow-up. However, if the likelihood of collapse is high, it may be advisable to recommend early surgery for such patients. Many potential predictors have been identified and proposed for predicting late reduction loss. However, to the best of our knowledge, no report exists on whether the bone density of the local bone correlates with the loss of reduction performed in the distal radius. This study analyzed the local bone mineral density (BMD) of the forearm for the first time as a factor of reduction loss in distal radius fracture. The result of our study showed that forearm BMD was not a valuable prognostic factor for reduction loss in distal radius fractures. Initial dorsal comminution and ulnar variance rather than forearm BMD should be considered when deciding which patients need surgery. The findings of this study provide a novel concept on the association of local bone conditions with the prognosis of fractures treated conservatively.

## Data Availability

The datasets generated during and/or analysed during the current study are available from the corresponding author on reasonable request.
